# Temporal activity patterns of layer II and IV rat barrel cortex neurons in healthy and injured conditions

**DOI:** 10.14814/phy2.15155

**Published:** 2022-02-23

**Authors:** Thomas F. Burns, Ramesh Rajan

**Affiliations:** ^1^ Biomedicine Discovery Institute Monash University Victoria Australia

**Keywords:** barrel cortex, electrophysiology, inhibition, temporal coding, traumatic brain injury

## Abstract

Neurons are known to encode information not just by how frequently they fire, but also at what times they fire. However, characterizations of temporal encoding in sensory cortices under conditions of health and injury are limited. Here we characterized and compared the stimulus‐evoked activity of 1210 online‐sorted units in layers II and IV of rat barrel cortex under healthy and diffuse traumatic brain injury (TBI) (caused by a weight‐drop model) conditions across three timepoints post‐injury: four days, two weeks, and eight weeks. Temporal activity patterns in the first 50 ms post‐stimulus recording showed four categories of responses: no response or 1, 2, or 3 temporally‐distinct response components, that is, periods of high unit activity separated by silence. The relative proportions of unit response categories were similar between layers II and IV in healthy conditions but not in early post‐TBI conditions. For units with multiple response components, inter‐component timings were reliable in healthy and late post‐TBI conditions but disrupted by injury. Response component times typically shifted earlier with increasing stimulus intensity and this was more pronounced in layer IV than layer II. Surprisingly, injury caused a reversal of this trend and in the late post‐TBI condition no stimulus intensity‐dependence differences were observed between layers II and IV. We speculate this indicates a potential compensatory mechanism in response to injury. These results demonstrate how temporal encoding features maladapt or functionally recover differently in sensory cortex after TBI. Such maladaptation or functional recovery is layer‐dependent, perhaps due to differences in thalamic input or local inhibitory neuronal makeup.

## 1 INTRODUCTION

Diffuse traumatic brain injury (TBI) can be caused by hypoxia, meningitis, edema, or rapid fluid movement in the brain, like those caused by rapid acceleration/deceleration or rotational forces in sporting or motor vehicle accidents (Di et al., 2016; Granacher, 2003; Langlois et al., 2006; Namjoshi et al., 2013). TBI is associated with increased risks of depression (Kreutzer et al., 2001) and epilepsy (Liesemer et al., 2011; Yeh et al., 2013), likely caused by damage to and/or reorganization of inhibitory microcircuits (Cohen et al., 2007; Gaetz, 2004; Hunt et al., 2010; Povlishock & Katz, 2005; Reeves et al., 1997; Werner & Engelhard, 2007). The specific loss and damage of axons and cells after diffuse TBI will differ between individuals and progress at different rates. Subsequent “functional recovery” (i.e., gradual improvement in behavioral assessment outcomes) is typically slow and generally does not return to pre‐injury functional levels, despite multifaceted and interacting regenerative processes being triggered post‐injury (Carron, Alwis, et al., 2016; Nudo, 2013). One hypothesis (Alwis et al., 2012, 2013; Carron, Alwis, et al., 2016; Greer et al., 2013) is that maladaptations of the recovering cortex, like potential synaptic changes between particular cell types due to specific cell type(s) death and circuit remapping, cause disruption to microcircuit function in such ways that cause excitation‐inhibition changes. This would partially explain damage and (functional) recovery differences observed between cortical layers and cell types (Cantu et al., 2015; Carron, Alwis, et al., 2016; Carron, Yan, et al., 2016). However, how such broad changes affect the encoding of sensory stimuli in sensory cortices during and after injury is not completely understood.

Multi‐unit activity from extracellular physiological recordings from sensory cortex can be used to directly measure and compare neural activities in terms of mean amplitude over stimulus presentation, response onset delay, or the delay to maximum response (Allitt, Iva, et al., 2016; Maravall et al., 2007). Such methods have been used to study pooled neural population responses in TBI barrel cortex models (Allitt, Iva, et al., 2016; Allitt, Johnstone, et al., 2016; Alwis et al., 2012, 2013, 2016; Alwis & Rajan, 2014; Carron, Alwis, et al., 2016; Carron, Yan, et al., 2016; Johnstone et al., 2013, 2014, 2015; Yan et al., 2013). Barrel cortex is an especially rich sensory area to study due to its ecological importance for rodents and the ability interpret and connect results to the significant number of past studies (Burns & Rajan, 2021). It is conveniently arranged into primary cortical columns which process the sensory information from individual whiskers from the rodent face, and the arrangement of these columns in barrel cortex maps to the arrangement of whiskers on the face. In this study, we re‐analyze data from such studies on a unit‐by‐unit basis, identifying and quantifying individual units’ temporal activity patterns in response to one basic and one naturalistic stimulus in healthy and perturbed states.

For four reasons, we here focus only on layers II and IV:(1) layers II and IV have a very different proportions and types of interneurons (Markram et al., 2004), which are known to be affected differently by diffuse TBI (Carron, Alwis, et al., 2016); (2) layer IV is the main input layer and layer II is one of the first and most sophisticated signal processing layers in barrel cortex (Burns & Rajan, 2021); (3) data from TBI‐affected rats consistently show more superficial layers have medium‐ and long‐term changes in their excitability compared to deeper layers (Allitt, Iva, et al., 2016; Johnstone et al., 2013); and (4) recordings of layers II and IV have been the most frequent in past studies of TBI barrel cortex. Future studies may extend our analyses to other layers. Although we do not perform waveform analysis or otherwise attempt to identify units as “fast spiking” or “regular spiking,” in relation to reason (1) above, we expected units from layer II to show greater perturbations to their temporal responses properties than layer IV given interneurons’ strong influence in various temporal coding schema in barrel cortex (Burns & Rajan, 2021; Markram et al., 2004).

Our analyses allow us to observe how temporal activity patterns transform between cortical layers IV and II and, when interpreted with knowledge of the neuroanatomy, indicate how sensory information is processed in these layers of barrel cortex in healthy and perturbed states. Such comparisons may help to establish the importance of specific interneuron subtypes, microcircuits, and the maladaptations relevant to TBI pathophysiology. We also present a novel graph theoretic method for studying temporal activity patterns of neurons.

## 2 METHODS AND MATERIALS

### 2.1 Electrophysiological database

We collated electrophysiological data from previous studies (Allitt, Iva, et al., 2016; Allitt, Johnstone, et al., 2016; Alwis & Rajan, 2013, 2014; Alwis et al., 2012, 2016; Carron, Yan, et al., 2016; Johnstone et al., 2013, 2014, 2015; Yan et al., 2013). The database is composed of data from 36 male Sprague‐Dawley rats, across three time‐points after traumatic brain injury (TBI) or sham surgery: four days (sham = 4, TBI = 6), two weeks (sham = 4, TBI = 9), and eight weeks (sham = 5, TBI = 8). TBI was caused by a modified version of the weight‐drop impact acceleration method (Foda et al., 1994; Hellewell et al., 2010), which aims to make the primary injury a diffuse TBI. A hallmark of this type of injury is that it does not typically result in any sizeable lesion in cortex, making it difficult injury to simply diagnose for clinicians and grossly measure for researchers. An impact velocity of 6.15 m/s was used to model a severe injury (Yan et al., 2013). At different timepoints after the injury, animals underwent terminal surgery where they were anesthetized with 5% halothane and tracheomatized to maintain anaesthesia at 0.5%–3% and allow continuous ventilation. Body temperature was maintained at 37–38°C. Depth of anaesthesia was monitored using ECG/EMG recordings from forepaw musculature, hind paw pinch, and palpebral reflexes. A craniotomy 5 mm in diameter was performed over the right barrel cortex (2 mm caudal to bregma; 6 mm lateral to the midline) and a microelectrode (2–4 MΩ; FHC, Bowdoinham, ME) was used to record from a single cortical column with the same principal whisker (PW). This PW was identified by manually stimulating individual whiskers with a toothpick until consistent, strong drive was found for a single whisker. The PW was then threaded with a computer‐controlled motor lever arm system to present precise stimuli protocols by deflecting the whiskers in the dorsal‐ventral direction, perpendicular to the face. Output signals from the microelectrode were amplified and bandpass‐filtered from 0.3 to 10 kHz. The microdrive controlling the depth of the inserted microelectrode was zeroed at the pia and advanced to different cortical depths from the pia. So as to limit the effects of the anaesthesia over time, some experimental recordings were from deep to superficial cortical depths whereas others were from superficial to deep (and in both cases moving in increments of 50 µm or 100 µm).

Some animals in the database underwent behavioural assessments for sensorimotor function and/or were studied using histological techniques post‐mortem. We were not able to systematically analyze these behavioural and histological data in this larger, combined dataset due to differences or absences in the use of these techniques among the animals under study. This study therefore focussed on analysis of the electrophysiological data, which was consistent in method across all animals. All experiments were conducted in accordance with the Code of Practice for the Care and Use of Animals for Scientific Purposes (National Health and Medical Research Council, Australia) and were approved by the Monash University Standing Committee on Ethics in Animal Experimentation. Further details of the injury model and experimental techniques can be found in in the original studies (Allitt, Iva, et al., 2016; Allitt, Johnstone, et al., 2016; Alwis & Rajan, 2013, 2014; Alwis et al., 2012, 2016; Carron, Yan, et al., 2016; Johnstone et al., 2013, 2014, 2015; Yan et al., 2013).

**FIGURE 1:**
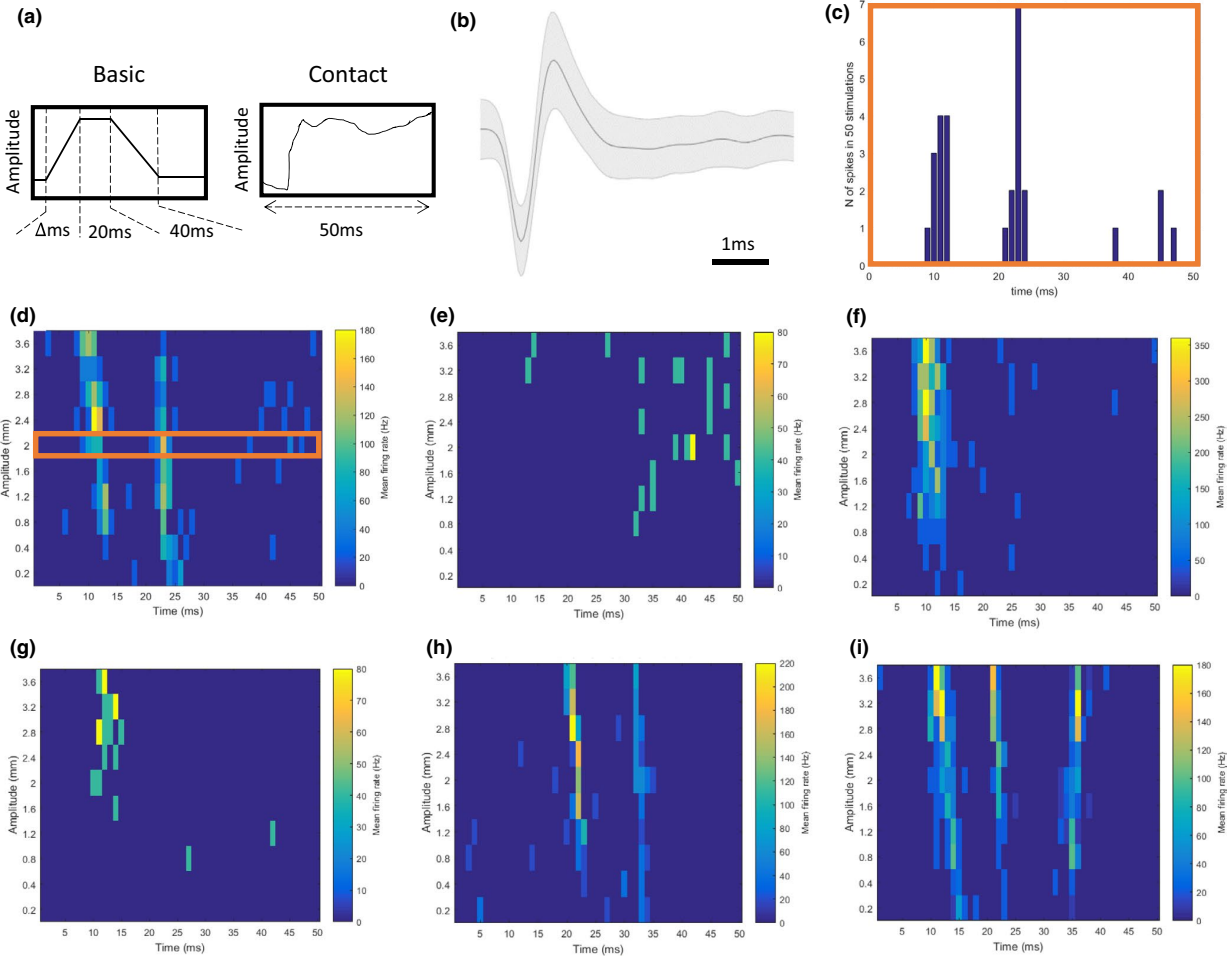
(a) Stimulus waveforms for “Basic” and “Contact,” the two tactile stimuli presented to the rodents which were studied in this project. (b) Representative waveform of an online‐sorted unit. The dark line indicates the mean normalized amplitude over 5 ms for all spikes sorted into this unit, with the shaded region showing the range of all spikes. (c) Example unit's PSTH in response to Contact at a maximum amplitude of 2 mm and (d) the PSTH converted into mean firing rates (Hz), represented in a heatmap across all amplitudes for the same unit. This unit response consists of two major components, one occurring at ~10 ms post‐stimulus onset and the second occurring at ~23 ms post‐stimulus onset across most amplitudes. (e–i) Example units responding to the Contact stimulus identified as having 0 (e), 1 (f–g), or ≥2 components (h–i) by the independent neural response components analysis (extended local maxima algorithm). Units with 0 components are classed as non‐responders and although they may show some tendencies to spike in general periods, for example, offset periods, these responses are not as reliable as those in units with ≥1 components. Units with ≥2 exhibit a wide diversity of component strengths and timings

From these studies, we analyzed online‐sorted units responding one of two stimuli (depicted in Figure 1a):

- “Basic”: A trapezoid stimulus had an on‐ramp of varying velocity (30, 60, 150, 250, or 400 mm/sec) to a distance of 3.6 mm from the rest whisker position, a hold period of 20 ms, and an off‐ramp over 40 ms back to rest (Allitt, Iva, et al., 2016; Alwis et al., 2012); and
- “Contact”: A naturalistic object contact stimulus which lasted a total of 100 ms and included an initial deflection followed by complex movement, reconstructed from high‐speed video‐tracking of awake behaving rats’ whiskers contacting an object and then brushing past it (Hartmann et al., 2003).

All analyses from this database used online‐sorted units, sorted by highly‐experienced electrophysiologists using the Cambridge Electronic Design Spike2 software. The relative amplitudes of spikes were visualized and shape‐based criteria (including rise time of the upstroke, the width of the action potential waveform, the size of the overshoot, etc.) were used to accept and reject units, ensuring units analyzed were highly reliable and of good quality. An example unit is shown in Figure 1b. Waveform analysis to functionally classify units (e.g., into categories of “fast spiking” and “regular spiking”) was not performed, although this would be an interesting although this would be an interesting future study to consider in combination with other techniques (e.g., histological) to confirm cells’ functional types and other anatomical information, and how this relates to their activity in TBI. The current database amounted to a total of 1210 units from layer II (150–300 µm from pia) or layer IV (750–1000 µm deep from pia) across both health and TBI conditions (summarized in Table 1). It should be noted that our classification of units into layer II and IV using depth estimation was not confirmed using histology, which represents a limitation of this data an analysis. Unit activity profiles were then quantified using established methods (Allitt, Iva, et al., 2016; Alwis et al., 2012).

**TABLE 1:** Number of online‐sorted units for each condition and layer for Basic and Contact stimuli

Condition	Layer II	Layer IV	Total
Basic stimulus			
Sham	88	142	230
TBI four‐days post‐injury (4 days TBI)	26	42	68
TBI two‐weeks post‐injury (2 weeks TBI)	39	65	104
TBI 8–12‐weeks post‐injury (8–12 weeks TBI)	55	88	143
Contact stimulus			
Sham	103	142	245
TBI four‐days post‐injury (4 days TBI)	39	66	105
TBI two‐weeks post‐injury (2 weeks TBI)	71	94	165
TBI 8–12‐weeks post‐injury (8‐12 weeks TBI)	63	87	150

Lastly, it is pertinent to note a few general limitations and confounding factors regarding this database. In general, diffuse TBI of the kind caused in these experiments causes a period of general period of hypoexcitation (especially in the supragranular layers) of barrel cortex. Many details are still under investigation, although we hope the current work helps to further address this question. Nevertheless, it is possible some of our results or interpretation thereof are confounded by unmeasured injury effects. Additional unmeasured effects may come from the anaesthesia. Furthermore, although rodent models are common animal model for biomedical and neuroscientific research, many of the structures in the rodent brain do not exist in humans, like barrel cortex, and the relationship of layers II and IV to primate granular layers is unclear. This means more translational work is necessary for the current work to have significant clinical impacts for human brain injury patients.

### 2.2 Unit activity profiles

Unit activity profiles were produced by calculating the peristimulus time histograms (PSTHs) for each unit at each velocity (for Basic) or amplitude (for Contact) across all the repetitions of that stimulus at that layer for that recording. Following the convention of and to allow for easier comparison to previous studies, spikes were counted in 1 ms bins up until 50 ms post‐stimulus onset. We then manually examined unit activity profiles in the form seen in Figure 1d–i. A diversity of response patterns existed. Most notably, some units appear to be non‐responsive whereas other units contain one or more response components, that is, one or more times at which the unit firing rate is markedly higher than the apparent baseline. Furthermore, some of these components appeared to be relatively stationary in time whereas others are non‐stationary and appear to occur earlier or later depending on the stimulus intensity—velocity (for Basic) or amplitude (for Contact).

### 2.3 Independent neural response components analysis

To objectively identify and quantify response components, an independent neural response components analysis was developed by extending the traditional local maxima algorithm. This analysis was done on a unit‐by‐unit basis and involved first identifying the local maxima in each PSTH for a given unit and noting the times at which these local maxima occurred. Local maxima were discarded if the mean firing rate for that 1 ms bin was <25% of max firing rate and the neighboring 1 ms bins had no spikes (or 0% of max firing rate). Local maxima occurring within 5 ms of each other were combined and the median 1 ms time bin between these bins was taken as a single local maximum. These combinations were performed iteratively, starting with the two local maxima nearest each other. This left an approximation of the times where mean firing rates in the PSTH were highest and at least >25% of max firing rate. This analysis was conducted independently across all stimulus velocities (for Basic) or amplitudes (for Contact). We then used the same combination method to take the median times at which local maxima occurred and combine them across amplitudes to approximate, for the whole unit, when the major components of activity occurred. This resulted in units being identified as having either none or one or more neural response components. Kolmogorov–Smirnov (KS) tests were performed to determine the probability of component timing distributions being significantly different between the layers and conditions. For units with multiple components, linear regressions were conducted to determine if the times at which independent components occurred within the same unit were related. We then plotted circle charts showing the relative proportions, for each layer and animal condition (and again independently for both Basic and Contact), of units which were non‐responders (having no response components) and for units which had one or more components. These relative proportions were then compared between layers and conditions using KS tests.

### 2.4 “Peakiness” and significance of components analysis

A concern about the definition of components using the independent neural response components analysis described above was that some components, while being “local maxima,” may or may not be statistically significant. Therefore, we used two methods to analyze how statistically valid the components we identified were: (i) a “peakiness” detection algorithm (Beveridge et al., 1989; Haralick & Shapiro, 1984; Jain et al., 1995); and (ii) a *t* test (which tests if the means of two populations are equal) comparing the components’ firing rates with the surrounding, non‐component firing rates. Both methods were used on a component‐by‐component basis for all units which had been identified as having at least one component. For these tests, we took the mean firing rates at the 5, 1 ms time bins occurring immediately before and after the identified component time. Together with the identified component time bin, this represented an 11 ms segment of mean firing rates for that unit, where the 6th ms bin represented the identified component time.

For the peakiness detection algorithm, we identified the local minima within the 11‐element segment and called this $g_{k}$. Then, $\mathrm{peakiness}=\frac{g_{i}+1}{g_{k}+1}$, where $g_{i}$ is the mean firing rate of the 6th ms bin. This provides us with a single number, “peakiness,” which described every component. This number varied on a scale of 0 to 2, where 0 represented that the identified component time had infinitely less firing than the local minima within ±5 ms from the component time, where 2 represented it having infinitely more, and where 1 represented it being equal. In practice, this meant that a peakiness value of 1.25 indicates that the mean firing rate at the identified component time was >25% than the surrounding mean firing rate minima within ±5 ms from the component time; a value of 1.5 would indicate >50%, and so on.

For the *t* tests, we took the 5th to 7th ms bin mean firing rates and called this segment the ‘component segment’. We then performed a one‐tailed two‐sample *t* test, comparing the mean firing rates of the component segment with the remaining 8, 1 ms bin mean firing rates of the same 11 ms‐long segment used in the peakiness detection algorithm.

### 2.5 Graph‐theoretic components analysis

**FIGURE 2:**
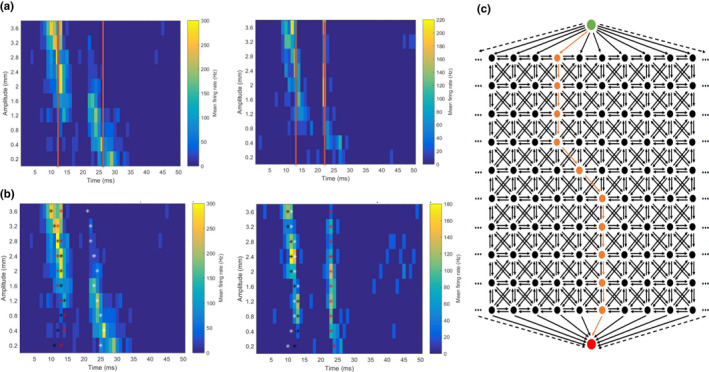
(a) Example unit activity profiles with red lines showing the component times approximated by the independent neural response components analysis. (b) Mean firing rates mapped in the amplitude‐time domain for two healthy layer IV units responding to the Contact stimulus. Coloured stars are plotted over the amplitude‐time bins along the three shortest, non‐ overlapping paths calculated via the graphic‐theoretic method described in the text (black = primary shortest path, red = secondary shortest path, white = tertiary shortest path). (c) Visualisation of the graph‐theoretic algorithm procedure to identify neural response components. Each black or orange vertex is equivalent to one ms bin at one amplitude in the PSTH matrix of a single unit. For example, for the Contact stimulus there are 50, 1 ms bins (only 10 are shown in this illustration) arranged in 10 rows of increasing amplitude. Each vertex is connected to its neighbours (in the time and intensity domains) via edges. Edges arriving at a vertex are weighted according to the firing rate at that vertex. A vertex with a high firing rate has a small edge weight and a vertex with a low firing rate will have a large edge weight. Using Dijkstra's shortest path algorithm, the shortest possible path (travelling along edges which accumulate the lowest weighted score, as determined by the sum of their weights) is found (orange path) along the path with high firing rates. The top green vertex and bottom red vertex are the start and goal vertices, a technical requirement of the Dijkstra's algorithm, and allow the path to start and finish at any vertex in the top and bottom rows, respectively

The independent neural response components analysis is effective at identifying single, stationary components. However, some components noticeably shifted in time as a function of velocity (for Basic) or amplitude (for Contact). The previous analysis therefore was unable to capture information about how stationary or non‐stationary these components were (e.g., Figure 2a). The non‐stationarity of components could be an important feature to measure since, for units which had more than one component, some components appeared to interact, for example, while ascending in amplitude one component might increase its activity while the other simultaneously and proportionally decreases (e.g., left panel of Figure 2a). Such interactions, if sufficiently separated in time, could be evidence of feedforward inhibition or disinhibition, known to be present, for example, in layer IV of barrel cortex (Burns & Rajan, 2021). To identify these features, we developed a novel graph‐theoretic analysis. Such analysis has the ability to more accurately and precisely capture timing information rather than aiming for a general, normalized analytic approaches such as latency, PSTH (peak‐widths), response duration, and firing rates.

PSTHs for each unit are treated as a matrix and transformed into weighted directed graphs. Vertices represent the neural activity at different velocities/amplitudes and times, and are connected to neighboring vertices via weighted, directed edges. Vertices receive weighted, directed edges from other vertices representing the neural activity at velocities/amplitudes of z±1 and at times of t±1 (including at combinations z±1,t±1), where z is the receiving vertex's velocity/amplitude and t is its time. These edges are made for all vertices that exist (Figure 2c). The edges are weighted according to the mean firing rate of the vertex they are directed towards so that edges leading to vertices with relatively high mean firing rates have lower weights than edges leading to vertices with relatively low mean firing rates, like so: $\frac{\mathrm{unit}\;\max\;\mathrm{firing}\;\mathrm{rate}}{\mathrm{vertex}\;\mathrm{mean}\;\mathrm{firing}\;\mathrm{rate}}$. A start and goal vertex are connected to the top and bottom rows of the PSTH matrix graph, respectively, and used to calculate the shortest path between these vertexes through the PSTH matrix graph using Dijkstra's shortest path algorithm (Dijkstra, 1959). This path preferentially travels through the vertices representing the highest mean firing rates (since edges leading to them had the least weight) but will ensure such vertices are as contiguous as possible, owing to the graph's structure. Once a shortest path is calculated, we re‐weight the edges leading to vertices in that shortest path with an arbitrarily large weight and re‐run the shortest path algorithm to find the secondary shortest path, which does not re‐visit any vertices from the primary shortest path. This process can be repeated as many times as desired and here we calculate only the first three paths. A copy of algorithm implemented in MATLAB is freely available at https://github.com/tfburns/graph‐theoretic‐identification‐of‐PSTH‐components.

Figure 2b shows an example of the three shortest, non‐overlapping paths through two units’ firing activity profiles, as mapped in the amplitude‐time space for the Contact stimulus. Once identified, the following features of these paths were calculated: (i) the median time bin which the path vertices represent; (ii) the mean firing rates at each path step (and how these change as a function of mean firing rates at the equivalent steps of other components’ within the same unit); and (iii) how stationary or non‐stationary the path is in time.

## 3 RESULTS

### 3.1 Component timings using the independent response components analysis

For both stimuli and layers there is a tendency for components to occur earlier, especially in layer IV, whereas layer II appears to have a more lognormal distribution of component times. The most common times for components to occur in layer II was typically a few ms after the most common times for components to occur in layer IV, however this difference was less pronounced for the Basic stimulus than for the Contact stimulus.

**FIGURE 3:**
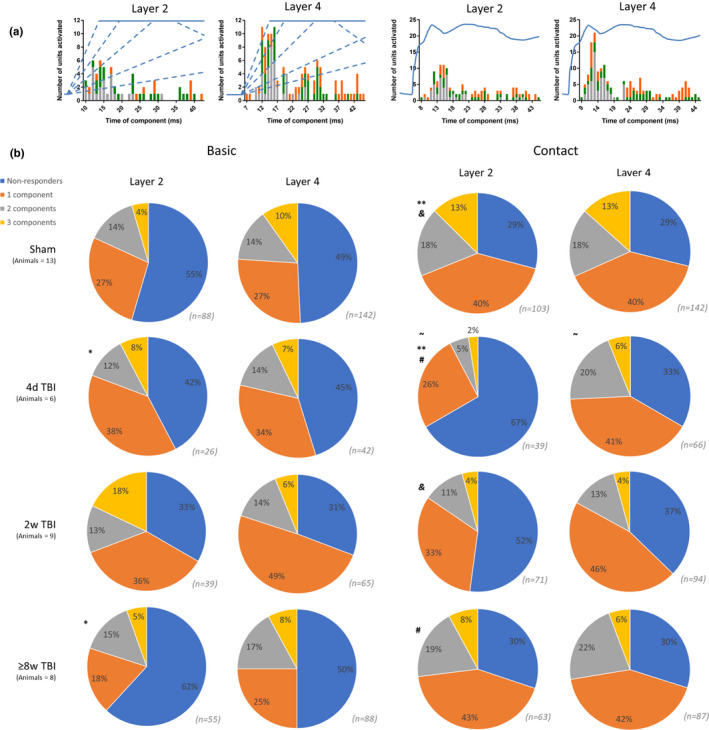
(a) Histogram of times of identified components occurring in units from sham animals for the Basic (two left panels) and Contact (right two panels) stimuli. Component times are shown from units with one component (grey), two components (green), and three components (orange). Plotted in blue is the relative amplitude over time for the Basic and Contact stimuli. These amplitudes have been aligned with the x‐axis to match what was being presented to the animal at the times these components are responding. In the case of the Basic stimulus, since five different on‐ramp velocities were tested the five respective amplitude trajectories have been plotted in dashed lines. The blue solid line at the top of the Basic stimulus depiction is the hold period, and the beginning of one off‐ramp period (from the 400 mm/sec on‐ramp velocity variation) is also depicted with a dashed line. Using these plots, it is possible to approximate what particular components might be representing in the stimuli. For instance, the component times in layer IV for Contact appear to have three separate modes: 9–18 ms representing the initial whisker deflection; 22–31 ms representing a second “bump” in the stimulus; and 39–44 ms representing where the stimulus dips to its lowest point since the original whisker deflection. (b) Proportions of units with different numbers of components (non‐responders = 0 components) for the Basic stimulus (left) and Contact stimulus (right) in layers II and IV with different health statuses. KS tests were conducted to determine if any proportions were significantly from one another. *, *p* < 0.05; ~ and &, *p* < 0.05; # and **, *p* < 0.001

By overlaying the stimulus waveforms on to the histograms of component times (Figure 3a), it is possible to qualitatively identify discrete modes or groups of components occurring together which may be representative of particular features. For instance, the component times in layer IV for Contact appear to have three separate modes: 9–18 ms representing the initial whisker deflection; 22–31 ms representing a second ‘bump’ in the stimulus; and 39–44 ms representing where the stimulus dips to its lowest point since the original whisker deflection. Further, the vast majority of the components in the second mode and all of the components in the third mode belong to units with two or three components, meaning that many units which respond to these later features of the stimulus also respond to earlier features. In comparison to layer IV, the component timings for Contact in layer II do not appear as well separated into distinct modes. Component timings in response to the Basic stimulus are more difficult to interpret, perhaps due to amplitude differences between the on‐ramp velocity conditions at particular time‐points. However, in the case of layer IV, all components occurring after 26 ms come from units which have previously responded to earlier stimulus features. Such components could therefore be signaling velocity or amplitude accumulation over slower on‐ramps, or the beginning or continuation of the holding period. Components which occur after 35 ms could also be associated with the beginning of the off‐ramp for the 400 mm/s on‐ramp velocity condition.

These results gave a helpful qualitative overview of the responses component timings relative to stimulus features. The most notable of these results being that single‐component responses typically occurred earlier in the stimulus windows, that is, as “stimulus on” or “stimulus start” responses.

### 3.2 Proportions of units with different numbers of components and their strength

Figure 3b shows the proportions of units with different numbers of response components to the Basic and Contact stimuli across layers and conditions. Across conditions, KS tests showed significant differences between the proportions across conditions in layer II but none in layer IV. Across layers, the only significant difference between the proportions was in the 4 days TBI condition for the Contact stimulus. Post‐TBI, layer 2 showed a dramatic reduction in the relative proportion of responsive units for the Contact stimulus. Responses to the Basic stimulus generally show a relative increase in the proportion of single‐component units in 4 days and 2 weeks TBI conditions across layers II and IV before normalization to sham levels in the 8–12 weeks TBI condition, where the number of multi‐component units become approximately as numerous as single‐component units but where the majority of units are non‐responsive. In contrast, responses to the Contact stimulus generally show a relative decrease in the proportion of responsive units post‐TBI, especially of multi‐component units and especially in layer II. However, like for the Basic stimulus, the distribution of units with different numbers of response components returns to a sham‐like distribution in the 8–12 weeks TBI condition.

Almost all components had a peakiness of ≥1.25 (94% of Basic and 92% of Contact components), indicating that averaged across all velocities or amplitudes, the mean firing rate was ≥25% higher at the identified component time than at the local minima within ±5 ms of the component. A large majority of components (76% of Basic and 79% of Contact components) had a peakiness of ≥1.5. Components from multi‐component units generally had lower peakiness values than single‐component units. This difference became more pronounced at higher thresholds, for example, for the Contact stimulus, 97% of single‐component and 87% of multi‐component units had ≥1.25 peakiness, whereas these numbers fell to 87% and 68%, respectively, at a peakiness threshold of ≥1.5.

The same differences were more pronounced when looking at the *t* tests performed using the mean firing rates on the components and the surrounding ±5 ms. While a vast majority of components had significantly higher mean firing rates than the surrounding ±5 ms at *α* = 0.05 (91% of Basic and 87% of Contact components), far fewer reached significance at *α* = 0.01 (46% of Basic and 52% of Contact components). The most significant differences were found for units with a single component, whereas few units with two or three components reach similar levels of significance; at *α* = 0.01 for Contact, 63% of single component units, 39% two component units, and 27% of three component units reached significance. Granted, 86% of multi‐component units reached significance at *α* = 0.05 (compared with 90% for single‐component units).

These results demonstrated the systematic changes in the proportions of unit response categories. At a high level, these changes show quite dramatic changes in early post‐TBI conditions followed by a gradual return to proportions which were functionally similar to healthy conditions in the late post‐TBI conditions.

### 3.3 Temporal regularity of components occurring within the same unit

**FIGURE 4:**
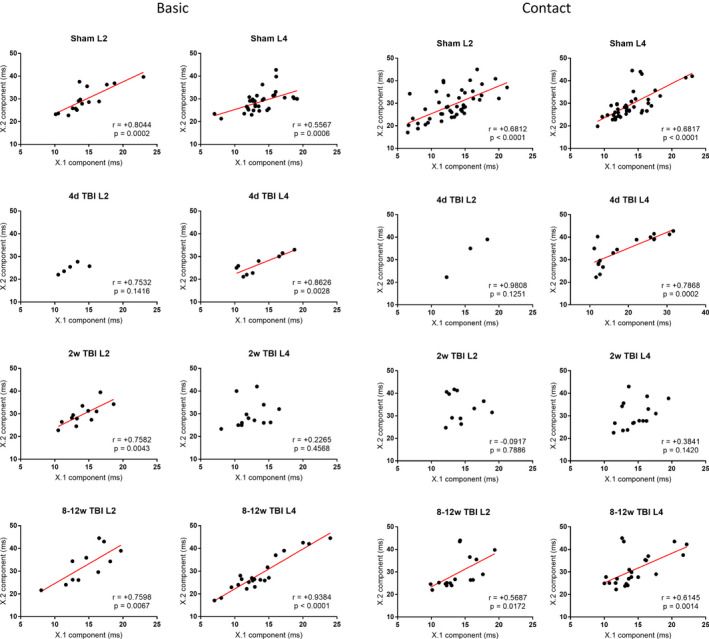
Linear regressions between the timings of first and second components of multi‐component units responding to the Basic (left) and Contact (right) stimuli, separated by layer and condition. Red lines show line of best fit where the relationship is significantly correlated. L2 and L4 = layers II and IV; X.1 and X.2 = first and second components. Linear regressions between the timings of first and second components of multi‐component units responding to the Contact stimulus, separated by layer and condition

For both Basic and Contact stimuli, a number of significant relationships were found between the timings of first and second components in multi‐component units. These relationships were particularly significant in the sham condition (*p* < 0.001 for both stimuli in both layers II and IV). Relationships in the 8–12 weeks TBI condition were also highly significant, especially in layer IV (*p* < 0.0001 for Basic and *p* = 0.0014 for Contact). However, there were irregular and insignificant timing relationships for both stimuli at the 4 days and 2 weeks TBI conditions (Figure 4).

To see how the relationships between these timings might reflect changes in the intracortical information flow after TBI, we calculated the y‐intercepts shown in Table 2. We notice that in both layers and for both stimuli there is a general increase in the standard errors (SEs) of the y‐intercepts, reflective of the higher scatter seen in Figure 4 for these relationships. Interestingly, considering the y‐intercepts from just the sham and 8–12 weeks TBI conditions shows that where the intercepts in one layer increase, intercepts in other layer decreases—and the relationships are opposite in the two stimuli. This may indicate temporal modulation in thalamic input to cortex via layer IV which is then being compensated for in layer II. The y‐intercepts (for those relationships which were significant), also gradually lowered across the TBI conditions—with one exception: the Contact layer IV y‐intercept increased after TBI. This exception is unusual given the Contact layer II y‐intercept decreased, where we might expect it to follow the layer IV trend.

**TABLE 2:** Y‐intercepts (±SEs) for linear regressions between the timings of first and second components of multi‐component units responding to the Basic and Contact stimuli in layers II and IV, as shown in Figure 4

Condition	Layer II	Layer IV
Basic stimulus		
Sham	9.459 ± 4.103	16.26 ± 3.393
TBI four‐days post‐injury (4 days TBI)	*# 13.28 ± 5.899*	10.37 ± 3.678
TBI two‐weeks post‐injury (2 weeks TBI)	9.06 ± 5.682	*# 22.49 ± 9.354*
TBI 8–12‐weeks post‐injury (8–12 weeks TBI)	7.271 ± 7.403	4.711 ± 2.052
Contact stimulus		
Sham	12.97 ± 2.618	8.248 ± 3.507
TBI four‐days post‐injury (4 days TBI)	*# −11.63 ± 8.814*	21.05 ± 2.887
TBI two‐weeks post‐injury (2 weeks TBI)	*# 37.63 ± 13.38*	*# 15.47 ± 9.723*
TBI 8–12‐weeks post‐injury (8–12 weeks TBI)	7.877 ± 8.245	12.15 ± 5.265

Y‐intercepts from insignificant regressions are marked #.

Like in the proportions of unit response categories (i.e., at the population level), these results at the individual unit level showed quite dramatic changes in early post‐TBI conditions followed by a gradual return to component timings which were functionally similar to healthy conditions in the late post‐TBI conditions.

### 3.4 Relationship between graph‐theoretic and independent neural response components analyses

Comparisons between the graph‐theoretic and independent neural response components analyses were only possible for units with one component. These relationships, where significant, are all positive. In the case of layer II, for the sham and 4 days TBI conditions the mapping is close to 1:1 between the times obtained for the same components from the two analyses. However, at the 2 weeks and 8–12 weeks TBI conditions this 1:1 mapping breaks down. In the case of layer IV, there is a weak positive correlation between the times obtained from the two methods, but this remains significant and relatively constant across animal conditions. Overall, the median path times tend to occur earlier than the local maxima component times calculated for the same unit components. In summary, this suggests the graph‐theoretic analysis is more sensitive to accurate timing information within unit response profiles whereas the neural response components analysis was more sensitive to precise overall timing of the response components.

### 3.5 Non‐stationarity of components

**FIGURE 5:**
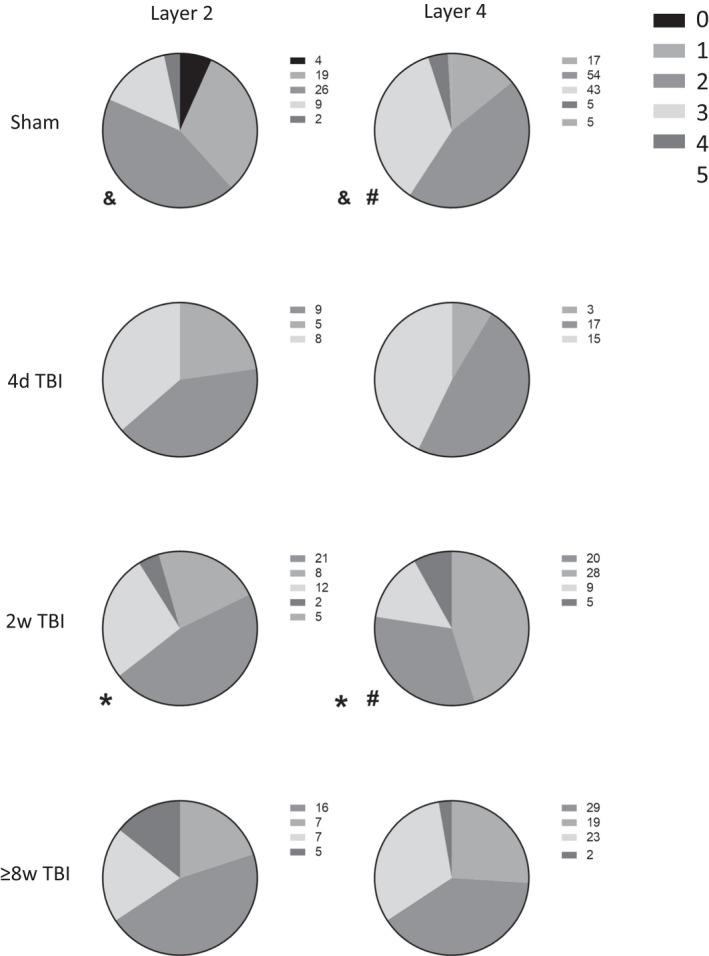
Proportions of units with different amounts of non‐stationarity (0–5 ms, indicated by the key top‐right). Significantly different proportion pairs are marked with symbols and *p*‐values given. &, *p* = 0.0187; #, *p* = 0.0008; *, *p* = 0.0401

Figure 5 shows the non‐stationarity of components (how much components “shifted” in time across different stimuli variations) for the Contact stimulus, separated by layer and condition. We can see that very few components are stationary, with most components moving across one or more ms as the stimulus varies in amplitude, the most common degree being 2 and 3 ms. This indicates that the function of microcircuitry controlling these temporal features are amplitude‐dependent.

Across the layer II conditions, most components move 2–3 ms, with a few being stationary in the sham condition and a minority across all conditions moving >3 ms. However, this minority of less stationary components seems to proportionally increase in TBI conditions compared to sham, although this trend in insignificant. What is significant are the of the differences between layer II and IV for the sham and 2 weeks TBI conditions. In the case of sham, layer IV components are much less stationary than layer II components, for example, only nine components had a movement of 3 ms in layer II, whereas in layer IV there were 43 components. Conversely, for the 2 weeks TBI condition, the significant difference between layers II and IV is due to a proportional increase in more stationary components—in layer II there were eight components with a movement of 2 ms, whereas in layer IV there were 28 components. This shift towards more stationary components also made the layer IV 2 weeks TBI condition distribution significantly different to the layer IV sham condition, which proportionally had more non‐stationary components. These results suggest that the amplitude‐dependent microcircuit properties controlling these temporal features may cause these changes in layers II and IV. Mechanistically, we speculate that we find components are more “mobile” in layer IV than layer II due to a layer IV having more direct thalamocortical inputs than layer II or layer II having more interneurons which serve to lessen these amplitude‐dependent effects. Most likely, it is some combination of these mechanisms.

Like results for the proportion of unit response types and intra‐unit timing relationships of components, these results further point to a significant change in early post‐TBI conditions before a return to a distribution which functionally similar to healthy conditions in the late post‐TBI condition. The exception in this case, however, is that differences appear slightly later, at the 2‐week timepoint, but not at the 4‐day timepoint.

### 3.6 Relationships between components’ mean firing rates

Across all layers and conditions there were many significant, positive correlations between the mean firing rates along component paths. Many of these paths are likely tracing segments of the same components, however it is also possible that these paths trace separate components which are affected by stimulus variations in a similar manner, for example, Figure 1d–i, bottom‐right panel. Unfortunately, we do not find any significant (at *α* = 0.05), negative correlations, as we might expect from a unit like that shown in Figure 2b, left panel. However, the majority of paths were not significantly correlated, positively or negatively, and this may be due to the low statistical power resulting in a higher likelihood of type II errors. Where we do see weak negative correlations, we notice that the sham condition generally contains more units with such relationships than the TBI conditions (especially for the 4 days TBI and 2 weeks TBI conditions). In the 8–12 weeks TBI condition, these weak negative correlations appear more frequently and comparable to the sham condition in this respect. This suggests that, if such results are indicative of cells with components which have oppositely‐weighted amplitude‐dependence (e.g., Figure 2b, right panel), such cells are less frequent in the early‐to‐mid functional recovery stages of TBI. This indicates such cells may be susceptible to maladaptation as a result of TBI. In summary, these results further added to evidence of functional changes in early post‐TBI conditions which become less pronounced or otherwise statistically similar (functionally) to health conditions in late post‐TBI conditions.

## 4 DISCUSSION

We have shown in a large database of online‐sorted units from layers II and IV of barrel cortex, diffuse TBI causes layer‐dependent functional maladaptations which affect the timing and number of response components. Multi‐component responsive units in healthy and late post‐TBI conditions have reliable inter‐component timing relationships, but not in early post‐TBI conditions. We introduced a graph‐theoretic method to characterize the shifting towards earlier post‐stimulus onset responses with increasing stimulus intensity, which was more pronounced in layer IV than layer II in the healthy condition. Immediately after TBI, this trend reversed and at 8‐12 weeks post‐TBI no stimulus intensity‐dependence differences were observed between layers II and IV. We speculate this potential compensatory mechanism in response to injury may be caused by differences in thalamic input or local inhibitory neuronal makeup.

### 4.1 Temporal activity patterns in healthy units

In healthy conditions, layer II units largely reflect the same sorts of temporal activity patterns as are seen in layer IV and in almost the same proportions. This is slightly surprising given the layers serve markedly different functions (Burns & Rajan, 2021)—the main layer IV excitatory output is to layers II and III whereas the layer II excitatory output is laterally to other parts of layer II (typically over several barrel columns), to other cells in layers III and V, and to secondary somatosensory and motor cortices (Aronoff et al., 2010; Feldmeyer et al., 2006). However, although the temporal activity patterns may be similar between layers II and IV, such patterns are not necessarily (and in fact unlikely to be) processed similarly. For example, the diverse inhibitory makeup of layer II may make transformation of input timing easier than complete silencing or splitting of arriving layer IV afferents, which would also suggest sensory processing in layer II barrel cortex is significantly temporal.

For both the Basic and Contact stimuli there were highly significant (*p* < 0.001) relationships between the times of components within individual layers. However, the y‐intercepts of the slope of the regression line between component timings for each layer were dissimilar, and the direction of difference between the layers was opposite between the stimuli (Table 2). This suggests that the way(s) layer II transforms temporal activity patterns from layer IV is stimuli‐dependent. In both cases, the layer II y‐intercept moves in the direction closer to ~10 ms relative to the layer IV y‐intercept. This might reflect an optimal inter‐component timing for layer II for processing of higher features, whereas layer IV, not needing to perform such processing and primarily acting to amplify the thalamic signal and distribute it to other cortical layers (Cowan & Stricker, 2004; Feldmeyer, 2012; Staiger et al., 2004), is capable of producing a wider variety of inter‐component timings (perhaps courtesy of its computationally simpler task). The initial inter‐component timings are also likely different for the two stimuli due to inherent differences in the stimuli themselves, for example the Basic stimulus had a constant peak amplitude (but variable on‐ramp time) whereas the Contact stimulus had varied peak amplitudes (but constant on‐ramp time). Notably, however, a large majority of the components occurred during the initial stimulus onset period, which appears to be a common response characteristic in other sensory systems (Nencini & Ivanusic, 2017). For both stimuli, the first components generally occur slightly later in layer II than in layer IV, which matches with the classical view of sensory information arriving at layer IV and then being transmitted up to layers II and III. Mechanistically, we could speculate that differences in timings between the components within individual unit components may be due to differences in inhibitory makeup between the layers, as well as the difference in thalamic input.

### 4.2 Temporal activity patterns in TBI units

Proportions of unit response profiles and their changes post‐TBI were quite different between the two stimuli: for the Basic stimulus there were more non‐responsive units and almost no changes post‐TBI whereas for the Contact stimulus there were initially fewer non‐responsive units and many changes post‐TBI. Although, in layer II, there was a general increase in non‐responsive units in early post‐TBI for both stimuli and then a general decrease in late post‐TBI, which may be reflective of the tendency of more superficial layers to experience greater functional and anatomical damage in TBI. The differences between the two stimuli may reflect the more complex, naturalistic nature of the Contact stimulus (Hartmann et al., 2003). Common between both stimuli, however, was a general reduction in the number of multi‐component units in the post‐TBI conditions, even after 8–12 weeks. If multi‐component units are responsible for processing more complex sensory information than fewer‐ or single‐component units, it is possible this reflects a permanent reduction in sensory processing capacity and associated cognitive deficiencies caused by TBI (Cantu et al., 2015; Ding et al., 2011; Johnstone et al., 2014).

Although the proportions of unit response profiles may have changed due to TBI, component times returned at 8–12 weeks post‐TBI to reliable inter‐component timing relationships like those seen in the healthy condition (see y‐intercepts in Table 2). Nevertheless, these results suggest stimuli‐dependent changes occurring in the timing between components, which agrees with the previously discussed sham timing data. This suggests either some considerable changes in the timing of thalamic efferent activity arriving in layers II and IV or TBI‐induced (possible compensatory) mechanisms. Given the primary input to layer II is via layer IV, it seems reasonable to expect if this was purely a cortical compensatory effect, the y‐intercept change should be immediate for layer IV at the 4 days TBI condition for both stimuli and would remain constant (whatever the direction of change), whereas the layer II y‐intercepts would slowly shift, indicating compensation in response to perturbed layer IV input. Instead, we find the y‐intercept is not constant across the TBI conditions in layer IV and instead slowly changes (along with layer II), suggesting a possible mixture of changes in thalamic input and intra‐cortical compensatory mechanisms (and it is possible this mixture is integrated via thalamocortical interactions in deeper layers). Possible intra‐cortical compensatory mechanisms may include gross anatomical changes such as the severing of feedforward inhibitory or excitatory synapses in layer II or slight but critical changes in spike timing leading to failure of previously expected feedforward signals (or, oppositely, success of previously unexpected feedforward signals).

Units responding to the Contact stimulus in layer IV of the 2 weeks TBI condition distribution had significantly (*p* = 0.008) fewer non‐stationary components compared to the sham condition. A similar, but less significant (*p* = 0.035) relationship was also found for the Basic stimulus. This further suggests a change in layer IV thalamic input. And while we do not find significant differences among the layer II non‐stationarity distributions for either stimulus, we do find significant differences for the Contact stimulus between layers II and IV for both the sham and 2 weeks TBI conditions. In the case of sham, layer IV components, they were much less stationary than layer II components, for example, only nine components had a movement of 3 ms in layer II, whereas in layer IV there were 43 components. Conversely, for the 2 weeks TBI condition, the significant difference between layers II and IV is due to a proportional increase in more stationary components—in layer II there were eight components with a movement of 2 ms, whereas in layer IV there were 28 components. This mis‐match between the direction of change for component timings agrees with our earlier results regarding multi‐component unit timings and their regressions’ y‐intercepts. It also supports the speculative idea that thalamic input in layer II, cortical compensatory mechanisms, or some combination thereof, is attempting to work “against” changes in layer IV activity, as driven by thalamic input. However, it is also possible that what we have been referring to as “compensatory” mechanisms (whether in cortex, via thalamic input, or corticothalamic interactions) are actually the malfunctioning of inhibitory microcircuits in layer II and such malfunctioning is the driver of these mis‐matches. If so, taken together with our results regarding the activity component distributions, it seems likely that inhibitory malfunction in TBI disrupts the timing of components more than the overall proportions and presence of such components. The fact that the non‐stationarity was more pronounced in layer IV than layer II for the health conditions (but not the late post‐TBI conditions) indicates, however, a permanent and significant deviation from healthy conditions. Mechanistically, we speculate this change is likely caused by damage or maladaptation in inhibitory microcircuits. We suggest future studies probe such circuits in the context of temporal response profiles of neurons in TBI conditions.

## 5 CONCLUSION

Collectively, along with the various stimulus‐dependent and layer‐dependent effects mentioned, these analyses suggest TBI disrupts normal temporal coding mechanisms in individual units of barrel cortex. Along with the previously‐established general reduction in inhibition after TBI (Carron, Alwis, et al., 2016), this emphasizes the importance of fine‐tuned inhibition on normal temporal coding mechanisms applied to sensory signals in layers II and IV. Simultaneously, it demonstrates that some unit profiles can appear functionally unchanged despite global excitatory‐inhibitory imbalance and microcircuit changes. Commutatively, however, these effects lead to an increase in non‐responsive units, changes in distributions of temporal activity patterns, and misalignment of the different components in multi‐component temporal response patterns. While some of these changes appear transient, others appear to persist long after TBI. It is likely some of these changes are partially responsible for the short‐ and long‐term behavioural and cognitive changes which occur after TBI. This warrants future work to more precisely identify the specific neuronal sub‐types involved in leading to these temporal activity differences, and also whether and where they arise (at different layers, and/or as transformations from thalamic activity). Future work may also consider tracking the behavioural or awake electrophysiological correlates of these temporal activity differences.

## CONFLICT OF INTEREST

The authors declare no conflicts of interest.

## AUTHOR CONTRIBUTIONS

TB and RR conceived of and conducted the research and wrote the paper.

## Data Availability

The data that support the findings of this study are available from the corresponding author upon reasonable request. A copy of the graph‐theoretic PSTH algorithm implemented in MATLAB is freely available at https://github.com/tfburns/graph‐theoretic‐identification‐of‐PSTH‐components.

## References

[phy215155-bib-0001] Allitt, B. J. , Iva, P. , Yan, E. B. , & Rajan, R. (2016). Hypo‐excitation across all cortical laminae defines intermediate stages of cortical neuronal dysfunction in diffuse traumatic brain injury. Neuroscience, 334. 10.1016/j.neuroscience.2016.08.018 27530700

[phy215155-bib-0002] Allitt, B. J. , Johnstone, V. P. A. , Richards, K. , Yan, E. B. , & Rajan, R. (2016). Progesterone exacerbates short‐term effects of traumatic brain injury on supragranular responses in sensory cortex and over‐excites infragranular responses in the long term. Journal of Neurotrauma, 33(4). 10.1089/neu.2015.3946 26258958

[phy215155-bib-0003] Alwis, D. S. , Johnstone, V. , Yan, E. , & Rajan, R. (2013). Diffuse traumatic brain injury and the sensory brain. Clinical and Experimental Pharmacology and Physiology, 40(7), 473–483. 10.1111/1440-1681.12100 23611812

[phy215155-bib-0004] Alwis, D. S. , & Rajan, R. (2013). Environmental enrichment causes a global potentiation of neuronal responses across stimulus complexity and lamina of sensory cortex. Frontiers in Cellular Neuroscience, 7, 124. 10.3389/fncel.2013.00124 23964199 PMC3737482

[phy215155-bib-0005] Alwis, D. S. , & Rajan, R. (2014). Environmental enrichment and the sensory brain: The role of enrichment in remediating brain injury. Frontiers in Systems Neuroscience, 8. 10.3389/fnsys.2014.00156 PMC4151031 25228861

[phy215155-bib-0006] Alwis, D. S. , Yan, E. B. , Johnstone, V. , Carron, S. , Hellewell, S. , Morganti‐Kossmann, M. C. , & Rajan, R. (2016). Environmental enrichment attenuates traumatic brain injury: Induced neuronal hyperexcitability in supragranular layers of sensory cortex. Journal of Neurotrauma, 33(11). 10.1089/neu.2014.3774 26715144

[phy215155-bib-0007] Alwis, D. S. , Yan, E. B. , Morganti‐Kossmann, M.‐C. , & Rajan, R. (2012). Sensory cortex underpinnings of traumatic brain injury deficits. PLoS One, 7(12). 10.1371/journal.pone.0052169 PMC3528746 23284921

[phy215155-bib-0008] Aronoff, R. , Matyas, F. , Mateo, C. , Ciron, C. , Schneider, B. , & Petersen, C. C. H. (2010). Long‐range connectivity of mouse primary somatosensory barrel cortex. European Journal of Neuroscience, 31(12), 2221–2233. 10.1111/j.1460-9568.2010.07264.x 20550566

[phy215155-bib-0009] Beveridge, J. R. , Griffith, J. , Kohler, R. R. , Hanson, A. R. , & Riseman, E. M. (1989). Segmenting images using localized histograms and region merging. International Journal of Computer Vision, 2(3), 311–347. 10.1007/BF00158168

[phy215155-bib-0010] Burns, T. F. , & Rajan, R. (2021). Sensing and processing whisker deflections in rodents. PeerJ, 9. 10.7717/peerj.10730 PMC7906041 33665005

[phy215155-bib-0011] Cantu, D. , Walker, K. , Andersen, L. , Taylor‐Weiner, A. , Hampton, D. , Tesco, G. , & Dulla, C. G. (2015). Traumatic brain injury increases cortical glutamate network activity by compromising GABAergic control. Cerebral Cortex [internet], 25(8), 2306–2320. http://www.ncbi.nlm.nih.gov/pubmed/24610117%5Cnhttp://www.pubmedcentral.nih.gov/articlerender.fcgi?artid = PMC4494035 10.1093/cercor/bhu041 PMC4494035 24610117

[phy215155-bib-0012] Carron, S. F. , Alwis, D. S. , & Rajan, R. (2016). Traumatic brain injury and neuronal functionality changes in sensory cortex. Frontiers in Systems Neuroscience [internet]. http://www.ncbi.nlm.nih.gov/pubmed/27313514%5Cnhttp://www.pubmedcentral.nih.gov/articlerender.fcgi?artid = PMC4889613 10.3389/fnsys.2016.00047 PMC4889613 27313514

[phy215155-bib-0013] Carron, S. F. , Yan, E. B. , Alwis, D. S. , & Rajan, R. (2016). Differential susceptibility of cortical and subcortical inhibitory neurons and astrocytes in the long term following diffuse traumatic brain injury. The Journal of Comparative Neurology, 1, 20–22. 10.1002/cne.24014 27072754

[phy215155-bib-0014] Cohen, A. S. , Pfister, B. J. , Schwarzbach, E. , Sean Grady, M. , Goforth, P. B. , & Satin, L. S. (2007). Injury‐induced alterations in CNS electrophysiology. Progress in Brain Research, 161, 143–169. 17618975 10.1016/S0079-6123(06)61010-8

[phy215155-bib-0015] Cowan, A. I. , & Stricker, C. (2004). Functional connectivity in layer IV local excitatory circuits of rat somatosensory cortex. Journal of Neurophysiology, 92(4), 2137–2150. 10.1152/jn.01262.2003 15201316

[phy215155-bib-0016] Di Virgilio, T. G. , Hunter, A. , Wilson, L. , Stewart, W. , Goodall, S. , Howatson, G. , Donaldson, D. I. , & Ietswaart, M. (2016). Evidence for acute electrophysiological and cognitive changes following routine soccer heading. EBioMedicine [internet]. http://linkinghub.elsevier.com/retrieve/pii/S235239641630490X 10.1016/j.ebiom.2016.10.029 PMC5264439 27789273

[phy215155-bib-0017] Dijkstra, E. W. (1959). A note on two problems in connexion with graphs. Numerische Mathematik, 1(1), 269–327. 10.1007/BF01386390

[phy215155-bib-0018] Ding, M. C. , Wang, Q. , Lo, E. H. , & Stanley, G. B. (2011). Cortical excitation and inhibition following focal traumatic brain injury. Journal of Neuroscience, 31(40), 14085–14094. 10.1523/JNEUROSCI.3572-11.2011 21976493 PMC3444158

[phy215155-bib-0019] Feldmeyer, D. (2012). Excitatory neuronal connectivity in the barrel cortex. Frontiers in Neuroanatomy [internet]. http://journal.frontiersin.org/article/ 10.3389/fnana.2012.00024/abstract PMC3394394 22798946

[phy215155-bib-0020] Feldmeyer, D. , Lübke, J. , & Sakmann, B. (2006). Efficacy and connectivity of intracolumnar pairs of layer 2/3 pyramidal cells in the barrel cortex of juvenile rats. The Journal of Physiology [internet], 575(Pt 2), 583–602. http://www.pubmedcentral.nih.gov/articlerender.fcgi?artid = 1819447&tool = pmcentrez&rendertype = abstract 10.1113/jphysiol.2006.105106 PMC1819447 16793907

[phy215155-bib-0021] Foda, M.‐A.‐E. , Marmarou, A. , Brink, W. , Foda, M.‐A.‐E. , & Brink, W. (1994). A new model of diffuse brain injury in rats: Part I: Pathophysiology and biomechanics. Journal of Neurosurgery [internet], 80(2), 291–300. http://thejns.org/doi/abs/ 10.3171/jns.1994.80.2.0291@col.2012.116.issue‐6%5Cnhttp://thejns.org/doi/abs/10.3171/jns.1994.80.2.0291@sup.2010.112.issue‐2%5Cnhttp://thejns.org/doi/abs/10.3171/jns.1994.80.2.0301 10.3171/jns.1994.80.2.0291 8283269

[phy215155-bib-0022] Gaetz, M. (2004). The neurophysiology of brain injury. Clinical Neurophysiology, 115(1), 4–18. 10.1016/S1388-2457(03)00258-X 14706464

[phy215155-bib-0023] Granacher, R. P. Jr (2003). Traumatic brain injury: Methods for clinical and forensic neuropsychiatric assessment (p. 501). CRC Press.

[phy215155-bib-0024] Greer, J. , Hånell, A. , Mcginn, M. , & Povlishock, J. (2013). Mild traumatic brain injury in the mouse induces axotomy primarily within the axon initial segment. Acta Neuropathologica, 126(1), 59–74. 10.1007/s00401-013-1119-4 23595276 PMC3691315

[phy215155-bib-0025] Haralick, R. M. , & Shapiro, L. G. (1984). Image segmentation techniques. Proceedings of SPIE—International Society for Optical Engineering, 548, 2–9.

[phy215155-bib-0026] Hartmann, M. J. , Johnson, N. J. , Towal, R. B. , & Assad, C. (2003). Mechanical characteristics of rat vibrissae: Resonant frequencies and damping in isolated whiskers and in the awake behaving animal. Journal of Neuroscience, 23(16), 6510–6519. 10.1523/JNEUROSCI.23-16-06510.2003 12878692 PMC6740620

[phy215155-bib-0027] Hellewell, S. C. , Yan, E. B. , Agyapomaa, D. A. , Bye, N. , & Morganti‐Kossmann, M. C. (2010). Post‐traumatic hypoxia exacerbates brain tissue damage: Analysis of axonal injury and glial responses. Journal of Neurotrauma [internet], 27(11), 1997–2010. http://www.liebertonline.com/doi/abs/ 10.1089/neu.2009.1245 20822466

[phy215155-bib-0028] Hunt, R. F. , Scheff, S. W. , & Smith, B. N. (2010). Regionally localized recurrent excitation in the dentate gyrus of a cortical contusion model of posttraumatic epilepsy. Journal of Neurophysiology, 103(3), 1490–1500. 10.1152/jn.00957.2009 20089815 PMC2887624

[phy215155-bib-0029] Jain, R. , Kasturi, R. , & Schunck, B. G. (1995). Machine vision (pp. 73–111). McGraw‐Hill.

[phy215155-bib-0030] Johnstone, V. P. A. , Shultz, S. R. , Yan, E. B. , O’Brien, T. J. , & Rajan, R. (2014). The acute phase of mild traumatic brain injury is characterized by a distance‐dependent neuronal hypoactivity. Journal of Neurotrauma, 31(22), 1881–1895. 10.1089/neu.2014.3343 24927383 PMC4224042

[phy215155-bib-0031] Johnstone, V. P. A. , Wright, D. K. , Wong, K. , O’Brien, T. J. , Rajan, R. , & Shultz, S. R. (2015). Experimental traumatic brain injury results in long‐term recovery of functional responsiveness in sensory cortex but persisting structural changes and sensorimotor, cognitive, and emotional deficits. Journal of Neurotrauma, 32(17), 1333–1346. 10.1089/neu.2014.3785 25739059

[phy215155-bib-0032] Johnstone, V. P. , Yan, E. B. , Alwis, D. S. , & Rajan, R. (2013). Cortical hypoexcitation defines neuronal responses in the immediate aftermath of traumatic brain injury. PLoS One, 8(5), e63454. 10.1371/journal.pone.0063454 23667624 PMC3646737

[phy215155-bib-0033] Kreutzer, J. S. , Seel, R. T. , Gourley, E. , Kreutzer, J. S. (2001). The prevalence and symptom rates of depression after traumatic brain injury: A comprehensive examination. Brain Injury [internet], 15(7), 563–576. http://www.tandfonline.com/doi/full/ 10.1080/02699050116884%5Cnhttp://www.ncbi.nlm.nih.gov/pubmed/11429086 10.1080/02699050010009108 11429086

[phy215155-bib-0034] Langlois, J. A. , Rutland‐Brown, W. , & Wald, M. M. (2006). The epidemiology and impact of traumatic brain injury a brief overview. The Journal of Head Trauma Rehabilitation, 21(5), 375–378. 10.1097/00001199-200609000-00001 16983222

[phy215155-bib-0035] Liesemer, K. , Bratton, S. L. , Zebrack, C. M. , Brockmeyer, D. , & Statler, K. D. (2011). Early post‐traumatic seizures in moderate to severe pediatric traumatic brain injury: Rates, risk factors, and clinical features. Journal of Neurotrauma, 28(1557‐9042 (Electronic)):755–762. 10.1089/neu.2010.1518 21381863

[phy215155-bib-0036] Maravall, M. , Petersen, R. S. , Fairhall, A. L. , Arabzadeh, E. , & Diamond, M. E. (2007). Shifts in coding properties and maintenance of information transmission during adaptation in barrel cortex. PLoS Biology, 5(2), 0323–334. 10.1371/journal.pbio.0050019 PMC1779810 17253902

[phy215155-bib-0037] Markram, H. , Toledo‐Rodriguez, M. , Wang, Y. , Gupta, A. , Silberberg, G. , & Wu, C. (2004). Interneurons of the neocortical inhibitory system. Nature Reviews Neuroscience [internet], 5(10), 793–807. 10.1038/nrn1519 15378039

[phy215155-bib-0038] Namjoshi, D. R. , Good, C. , Cheng, W. H. , Panenka, W. , Richards, D. , Cripton, P. A. , & Wellington, C. L. (2013).Towards clinical management of traumatic brain injury: A review of models and mechanisms from a biomechanical perspective. Disease Models & Mechanisms [internet], 6(6),1325–1338. http://www.pubmedcentral.nih.gov/articlerender.fcgi?artid = 3820257&tool = pmcentrez&rendertype = abstract 10.1242/dmm.011320 PMC3820257 24046354

[phy215155-bib-0039] Nencini, S. , & Ivanusic, J. (2017). Mechanically sensitive A δ nociceptors that innervate bone marrow respond to changes in intra‐osseous pressure. The Journal of Physiology, 13, 4399–4415. 10.1113/JP273877 PMC5491870 28295390

[phy215155-bib-0040] Nudo, R. J. (2013). Recovery after brain injury: Mechanisms and principles. Frontiers in Human Neuroscience [internet], 7, 1–14. http://journal.frontiersin.org/article/ 10.3389/fnhum.2013.00887/abstract PMC3870954 24399951

[phy215155-bib-0041] Povlishock, J. T. , & Katz, D. I. (2005). Update of neuropathology and neurological recovery after traumatic brain injury. Journal of Head Trauma Rehabilitation [internet], 20(1), 76–94. http://www.ncbi.nlm.nih.gov/pubmed/15668572 10.1097/00001199-200501000-00008 15668572

[phy215155-bib-0042] Reeves, T. M. , Lyeth, B. G. , Phillips, L. L. , Hamm, R. J. , & Povlishock, J. T. (1997). The effects of traumatic brain injury on inhibition in the hippocampus and dentate gyrus. Brain Research, 757(1), 119–132. 10.1016/S0006-8993(97)00170-4 9200506

[phy215155-bib-0043] Staiger, J. F. , Flagmeyer, I. , Schubert, D. , Zilles, K. , Kötter, R. , & Luhmann, H. J. (2004). Functional diversity of layer IV spiny neurons in rat somatosensory cortex: Quantitative morphology of electrophysiologically characterized and biocytin labeled cells. Cerebral Cortex, 14(6), 690–701. 10.1093/cercor/bhh029 15054049

[phy215155-bib-0044] Werner, C. , & Engelhard, K. (2007). Pathophysiology of traumatic brain injury. British Journal of Anaesthesia [internet], 99(1), 4–9. http://www.ncbi.nlm.nih.gov/pubmed/17573392%5Cnhttp://bja.oxfordjournals.org/content/99/1/4.full 10.1093/bja/aem131 17573392

[phy215155-bib-0045] Yan, E. B. , Johnstone, V. P. A. , Alwis, D. S. , Morganti‐Kossmann, M. C. , & Rajan, R. (2013). Characterising effects of impact velocity on brain and behaviour in a model of diffuse traumatic axonal injury. Neuroscience, 248, 17–29. 10.1016/j.neuroscience.2013.05.045 23735754

[phy215155-bib-0046] Yeh, C. , Chen, T. , Hu, C. , Chiu, W. , & Liao, C. (2013). Risk of epilepsy after traumatic brain injury: A retrospective population‐based cohort study. Journal of Neurology, Neurosurgery & Psychiatry [internet], 84(4), 441–445. http://www.ncbi.nlm.nih.gov/pubmed/23117492%5Cnhttp://jnnp.bmj.com/content/84/4/441 10.1136/jnnp-2012-302547 23117492

